# Intestinal Macrophages at the Crossroad between Diet, Inflammation, and Cancer

**DOI:** 10.3390/ijms21144825

**Published:** 2020-07-08

**Authors:** Greta Caprara, Paola Allavena, Marco Erreni

**Affiliations:** 1 Department of Experimental Oncology, IEO, European Institute of Oncology, IRCCS, 20139 Milano (Mi), Italy; greta.caprara@ieo.it; 2 Laboratory of cellular immunology, Humanitas Clinical and Research Center-IRCCS-, via Manzoni 56, 20089 Rozzano (Mi), Italy; paola.allavena@humanitasresearch.it; 3 Humanitas University, Department of Biomedical Sciences, via Rita Levi Montalcini 4, 20090 Pieve Emanuele-Milan, Italy; 4 Unit of Advanced Optical Microscopy, Humanitas Clinical and Research Center-IRCCS-, via Manzoni 56, 20089 Rozzano (Mi), Italy

**Keywords:** macrophage, diet, microbiota, inflammation, colorectal cancer, gut, tumor-associated macrophage, intestine

## Abstract

Intestinal macrophages are key players in the regulation of the oral tolerance, controlling gut homeostasis by discriminating innocuous antigens from harmful pathogens. Diet exerts a significant impact on human health, influencing the composition of gut microbiota and the developing of several non-communicable diseases, including cancer. Nutrients and microbiota are able to modify the profile of intestinal macrophages, shaping their key function in the maintenance of the gut homeostasis. Intestinal disease often occurs as a breakdown of this balance: defects in monocyte–macrophage differentiation, wrong dietary habits, alteration of microbiota composition, and impairment in the resolution of inflammation may contribute to the development of intestinal chronic inflammation and colorectal cancer. Accordingly, dietary interventions and macrophage-targeted therapies are emerging as innovative tools for the treatment of several intestinal pathologies. In this review, we will describe the delicate balance between diet, microbiota and intestinal macrophages in homeostasis and how the perturbation of this equilibrium may lead to the occurrence of inflammatory conditions in the gut. The understanding of the molecular pathways and dietary factors regulating the activity of intestinal macrophages might result in the identification of innovative targets for the treatments of intestinal pathologies.

## 1. Introduction

The gastrointestinal (GI) tract hosts the largest compartment of the immune system. As the gut is continuously exposed to foreign antigens, mononuclear phagocytes play a crucial role in maintaining intestinal homeostasis, discriminating between innocuous antigens and potential pathogens. The breakdown of this equilibrium leads to the formation of chronic inflammation, where an inappropriate immune response to commensal microbiota occurs, possibly predisposing to the onset of colorectal cancer. As documented by a variety of research studies on this topic, intestinal macrophages are known key elements in the regulation of intestinal inflammation, both in physiological and pathological condition.

In this review, we will provide an overview of the functions of gut macrophages and their protective activities during intestinal health and disease. We will first describe the origin of intestinal macrophage population, then we will discuss their role in gut inflammation and, subsequently, in the establishment and progression of colorectal cancer. In particular, we will inspect the connection between macrophages and intestinal microbiota, focusing on how diet can influence this interaction and, more in general, the inflammatory status of the gut. Finally, we will review current developments of new therapies targeting macrophages in inflammatory bowel diseases and colorectal cancer.

## 2. Origin of Intestinal Macrophages

Tissue macrophages are traditionally thought to derive from blood-circulating monocytes that originate from highly proliferative bone marrow (BM) precursors [1]. In most peripheral tissues, resident macrophages derive from embryonic precursors arising from the yolk sac or fetal liver, populate tissues before birth and, during adulthood, maintain themselves autonomously by in situ self-renewal [2,3]. The intestine represents an exception to this rule, being initially seeded by embryo-derived macrophages, which are, with age, replaced by circulating monocytes [4]. It is still debated whether a small contribution of embryonic-derived, self-maintaining intestinal macrophages persists in the adults; a recent study showed the concomitant presence of a self-renewal macrophages of embryonic origin and BM-derived monocytes [5]. CCR2-CCL2 axis is the major driver of monocyte recruitment in tissues, both in physiological and pathological conditions: in mice, genetic depletion of the *Ccr2* gene results in a reduced macrophage number in the gut, indicating that Ly6C^hi^ monocytes, whose egress from the bone marrow is defective in this model, are the main precursors of intestinal macrophages in the adulthood; in line with this observation, the adoptive transfer of Ly6C^hi^ monocytes is able to restore intestinal macrophage populations [6,7].

Once in the gut, monocytes differentiate into macrophages, in a process taking approximately 5–6 days, known as monocyte “waterfall” [6,8]. Briefly, Ly6C^hi^ CX3CR1^int^ MHCII^−^ (P1) monocytes enter the intestinal tissue and, progressively, acquire the expression of MHCII (P2), downregulate Ly6C^hi^ (P3), together with other proteins responsible for monocyte extravasation (including CCR2, CD62L, LFA-1 and VLA-1), and, finally, upregulate the expression of CX3CR1, giving rise to mature macrophages (P4) [9,10]. Of note, a similar process has been observed in the human intestinal mucosa, with CD14^hi^ CCR2^+^ CD11c^hi^ monocytes progressively differentiating into CD14^low^ CCR2^−^ CD11c^low^ macrophages [6,11,12].

As previously reported, the presence of a self-renewal macrophage within the gut is still debated. By longitudinal fate-mapping experiments and tissue-protected bone marrow chimeric mice, a subpopulation of long-lived macrophages, expressing CD4 and Tim4, has been identified in the intestinal wall, persisting up to 8 months. These cells are predominantly located in the muscularis layer and in the submucosa, while mucosal macrophages seem to be more prone to continuous turnover from BM-derived monocytes [5,13].

In addition to the chemokine axis CCR2-CCL2, monocyte recruitment can be induced by a “physiological level of inflammation”, generated by the exposure to commensal bacteria and to antigens ingested with the diet [14]. A number of studies underlined the key role of microbiota in shaping macrophage populations in the mucosa: it has been demonstrated that microbial colonization, particularly during weaning, induces changes in colonic macrophage compartment, while administration of broad spectrum antibiotics affects macrophage turnover. Accordingly, a reduced macrophage number can be found in the gut of germ-free mice [4,15].

## 3. Macrophages in Intestinal Homeostasis

### 3.1. Environmental Factors Shaping Intestinal Macrophage Homeostatic Function

**Figure 1:**
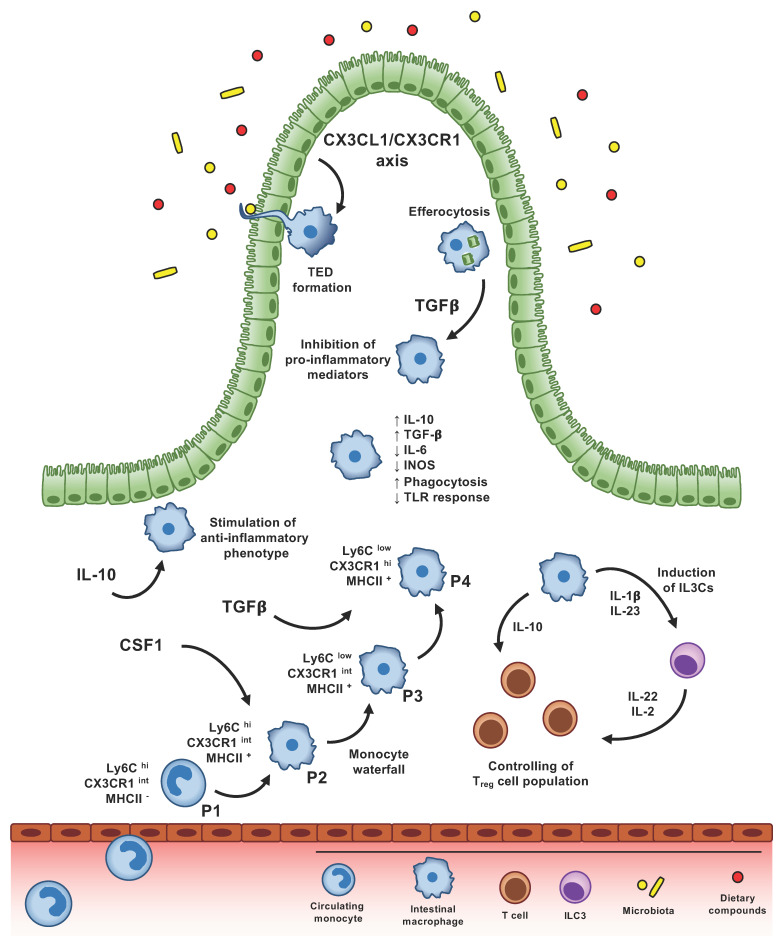
**Factors shaping intestinal macrophage phenotype and function.** Intestinal macrophages derive from circulating monocytes that, once in the gut, differentiate into macrophages in a process known as monocyte “waterfall”: Ly6C^hi^ CX3CR1^int^ MHCII^−^ (P1) monocytes enter the intestinal tissue, acquire the expression of MHCII (P2), downregulate Ly6C(P3), and, finally, upregulate the expression of CX3CR1, becoming mature macrophages (P4). In homeostatic condition, intestinal macrophages show a pro-resolving phenotype, characterized by an increased production of anti-inflammatory molecules, such as IL-10 and TGF-β, reduced expression of pro-inflammatory mediators, including IL-6 and iNOS, hypo-responsiveness to TLR stimulation, higher phagocytic activity and support of T_reg_ cell expansion. CSF1 is the primary cytokine involved in the differentiation and survival of gut macrophages, while TGFβ/TGFβ-receptor axis is fundamental for their terminal differentiation, modulating the expression of genes associated with their homeostatic phenotype. In addition, TGFβ is released by macrophages after efferocytosis and phagocytosis of apoptotic cells, inhibiting, through a paracrine/autocrine mechanism, the secretion of proinflammatory mediators by macrophages themselves. Response to IL-10 stimulation is crucial to induce the anti-inflammatory profile of gut macrophages. CX3CR1 expressing macrophages are able to control T_reg_ cell population, directly through the secretion of IL-10, or through the stimulation of ILC3. CX3CR1-macrophages, close to CX3CL1-expressing epithelial cells, extend trans-epithelial dendrites (TEDs) across the epithelial layer and capture soluble antigens or potential pathogens within the lumen.

Macrophages play a pivotal role in the maintenance of intestinal homeostasis. Differentiation from blood-circulating monocytes into macrophages is paralleled by the acquisition of a pro-resolving phenotype, characterized by an increased production of anti-inflammatory cytokines (such as IL-10), reduced secretion of pro-inflammatory molecules (such as IL-6 and iNOS), enhanced phagocytic activity, expression of scavenger receptors, and a reduced response upon Toll-like receptor (TLR) engagement [4,16]. However, exact factors and mechanisms that modulate their immune-modulatory phenotype are still under investigation (Figure 1).

#### 3.1.1. CSF1

CSF1 is the primary cytokine involved in the differentiation and survival of intestinal macrophages. Genetic deletion of *Csf1* gene, as well as the administration of anti-CSFR1 antibody, result in a reduced number of intestinal macrophages in mice [17,18]. In addition, it has been demonstrated that treatment with anti-CSF1R1 antibody results in an impaired differentiation of Paneth cells and reduction of Lrg5^+^ stem cells, influencing the development of the intestinal epithelium, including M and Goblet cells [19]. With regards to the maintenance of epithelial barrier, CD11b^+^ mucosal macrophages have been shown to be involved in the clearance of apoptotic intestinal epithelial cells via efferocytosis: phagocytosis of apoptotic epithelial cells is associated with an immunosuppressive transcriptional profile, including the downregulation of TLR2, preventing the occurrence of an inflammatory, autoimmune response [20].

#### 3.1.2. IL-10/IL-10R Axis

The IL-10/IL-10-receptor (IL-10R) axis plays a key role in the regulatory function of intestinal macrophages, both in mouse and humans. Disruption of this axis results in higher macrophage expression of pro-inflammatory mediators, as well as macrophage hyper-responsiveness to TLR engagement, leading to the occurrence of intestinal inflammation [21,22,23]. Accordingly, polymorphism in *IL10RA* and *IL10RB* genes is associated with the early onset of inflammatory bowel diseases (IBDs) in humans: of note, in vitro-differentiated macrophages from these patients robustly respond to TLR stimulation [24]. The exacerbation of the pro-inflammatory response in the absence of IL-10R can be also due to the failure in the downregulation pathway controlling macrophage activation, including STAT3, TREM-1, and STAT1, as well as in an increased chromatin accessibility to pro-inflammatory genes, which is normally restricted by IL-10-dependent epigenetic remodeling [22,25].

#### 3.1.3. CX3CR1-CX3CL1

The CX3CL1-CX3CR1 axis is crucial for colonic macrophage differentiation and function, as indicated by their high expression of CX3CR1 and their localization close to the CX3CL1-positive intestinal epithelial cells [26]. Absence of CX3CR1 expression by colonic macrophages results in reduced secretion of IL-10 and failure in supporting lamina propria T_reg_ cell expansion: accordingly, lack of CX3CL1-CX3CR1 signaling leads to a more sever dextran sulfate sodium (DSS)-induced colitis [27,28]. In addition, it has been demonstrated that the expression of IL-10Rα by CX3CR1^+^ macrophages is important for their anti-inflammatory profile in homeostatic conditions. Lack of macrophage response to IL-10 results in the expression of several pro-inflammatory mediators and the development of colitis in mice: in particular, IL-23 secretion by IL-10Rα-negative macrophages induces IL-22 release by ILC3 and Th17 T cells, leading to the occurrence of colitis [22,29]. Similarly, IL-23 and IL-1β production by CX3CR1^+^ macrophages induces ILC3 secretion of IL-22 and IL-2, thus controlling T_reg_ cell population [30,31]. In line with this observation, although in humans CX3CR1 expression by intestinal macrophages is reduced compared to mice, a missense mutation in the *CX3CR1* gene has been identified in patients affected by Crohn’s disease, in association with an impaired antifungal response and consequent increase of extra-intestinal inflammatory conditions [32]. CX3CR1^+^ macrophages have also been observed to form trans-epithelial dendrites (TEDs), able to cross the epithelial layer and capture soluble antigens or potential pathogens [33,34]. Recently, a role for tissue-resident CX3CR1^+^ macrophages has been also proposed in the maintenance of the intestinal vasculature: targeted depletion of this macrophage population results in morphological abnormalities in the submucosal vasculature and vascular leakage [5].

#### 3.1.4. TGFβ

The TGFβ/TGFβ-receptor axis is fundamental for the terminal differentiation of mucosal intestinal macrophages, modulating the expression of genes conventionally associated with their homeostatic profile, such as *CX3CR1*, *IL-10*, and *αvβ5* integrin [9]. Accordingly, TGFβ regulates the expression of the Runt-related transcription factor 3 (RUNX3), which is characteristic of intestinal macrophages [35]. Moreover, it has been proposed a role for the TGFβ/TGFβ-receptor axis in the control of macrophage turnover, by regulating their expression of CCL8 [9]. In addition, efferocytosis could induce TGFβ secretion by macrophages: of note, phagocytosis of apoptotic cells, in vitro, inhibits the production of pro-inflammatory mediators through an autocrine/paracrine mechanism involving TGFβ [36]. In humans, TGFβ-stimulation inhibits the pro-inflammatory response in blood-derived monocytes, through the downregulation of IL-6, IL-8, TNF, and IL-1β: anyway, this process seems not to occur in rodents, where colonic macrophages from *Tgfbr1*^−/−^ mice showed a reduced expression of TNF and IL-10, both in untreated and after LPS stimulation, when compared to their littermate controls [9].

### 3.2. Macrophages and Microbiota Crosstalk: The Impact of Diet

The mammalian GI tract is colonized by ~100 trillion symbiotic microorganisms, including bacteria, fungi, parasites, and viruses. This internal microbial community, which is primarily composed of bacterial cells (~99%), is referred to as the microbiota [37]. The microbiota engages a constant crosstalk with the intestinal epithelium, living in a state of symbiosis with the host’s body and exerting a significant impact on many physiological processes, including energy harvest, metabolism, immune response, immune system maturation, and regulation of neurological and cognitive development [38]. The gut microbiota plays a critical role in the development and function of the immune system, which, in turn, modulates composition and function of the microbiota itself. The constant exposure to commensal bacteria and diet-derived antigens leads to the formation of a mild inflammatory milieu in the intestinal mucosa. Because of their proximity to the intestinal epithelium, and consequent exposure to luminal content, mucosal macrophages display a more pro-inflammatory phenotype, compared to their muscularis counterpart. Nevertheless, they are equipped to be tolerogenic, allowing a tightly balance between pathogen clearance and the regulation of intestinal immune system, thus maintaining the integrity of the tissue [39]. Existing in such a microbe-rich environment, macrophages have acquired a functional hypo-responsiveness to the exogenous stimulations: in order to discriminate between pathogenic and commensal bacteria, it has been proposed that intestinal macrophages are able to recognize symbiotic microbial molecules, becoming active only when pro-IL-1β (whose regular production via macrophages is stimulated by microbiota) is cleaved into its mature form IL-1β, thus triggering the phagocytic process to kill invading pathogens [40]. In addition, the adherent bacterium *Clostridium butyricum* suppresses the anti-microbial program by inducing IL-10 macrophage secretion. Accordingly, depletion of microbiota, upon antibiotic administration, reduces IL-10 macrophage production, promoting Th1 cell increase [41,42]. Interestingly, it has been demonstrated a novel microbiota-dependent interaction between macrophages and RORγt^+^ ILC3: microbiota stimulates macrophages to produce IL-1β which, in turn, induces the release of CSF2 by ILC3, leading to the secretion of IL-10 by macrophages and, finally, the regulation of gut homeostasis via T_reg_ cell expansion [43].

**Figure 2:**
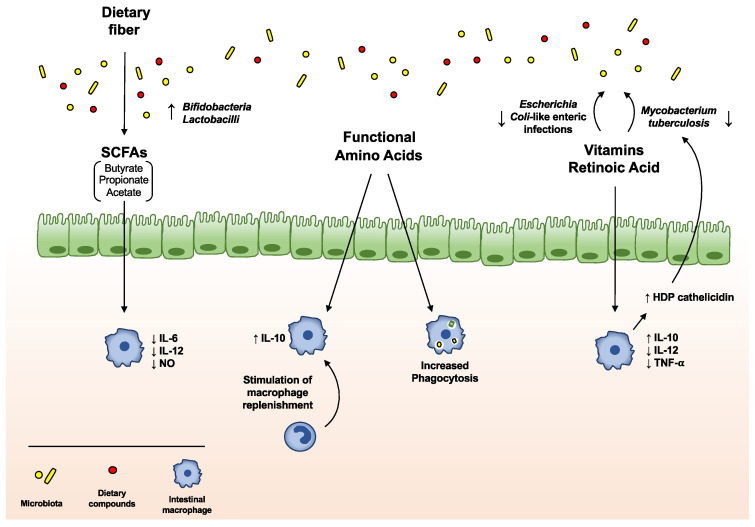
**Impact of diet on macrophage/microbiota crosstalk.** The microbial community regulates intestinal macrophage function via the release of specific compounds, often derived from the metabolism of dietary components. Dietary fiber is metabolized by intestinal microbes, leading to the production of the short-chain fatty acids (SCFAs) butyrate, propionate and acetate, which, in turn, promote the growth of specific beneficial microbiota species, including *Bifidobacteria* and *Lactobacilli*. In addition, these fermentation products modulate the immune response and macrophage activity, downregulating LPS-induced pro-inflammatory mediators, such as IL-6, IL-12, and NO. Dietary amino acids are able to regulate intestinal macrophage functions, increasing their phagocytic activity and stimulating the replenishment of intestinal macrophages, as well as their secretion of IL-10. Vitamins, in particular A and D, are involved in the control of intestinal macrophage functions. Vitamin A and its active metabolite retinoic acid (RA) attenuate intestinal inflammation in experimental models, reducing the synthesis of IL-12 and TNF-α, while increasing the production of IL-10, in LPS-stimulated macrophages. In addition, vitamin D suppresses the secretion of pro-inflammatory cytokines and directly stimulates the production of the host defense peptide (HPD) cathelicidin, required, in macrophages, for the anti-microbial activity against *Mycobacterium tuberculosis.* In addition, vitamin A and oral administration of RA are able to counteract the occurrence of *Escherichia coli*-like enteric and *M. tuberculosis* infections, respectively.

The microbial community may act on macrophage modulation via the release of specific compounds, often derived from the metabolism of dietary components (Figure 2). For instance, the anergic phenotype of intestinal macrophages is established through the delivery of short-chain fatty acids (SCFAs), derived by gut bacterial fermentation of dietary fibers, and aryl hydrocarbon receptor (AhR) ligands, originating from cruciferous vegetable indole derivatives [44]. Indeed, exposure of mouse macrophages to SCFA butyrate leads to the downregulation of LPS-induced pro-inflammatory mediators, such as IL-6, IL-12, and NO, restoring intestinal immune homeostasis [45,46]. Moreover, the combination of diet- and gut microbiota-derived indole derivatives with SCFAs regulates the susceptibility to intestinal inflammation in macrophages [16]. In the last years, an increasing role for enteral nutrients in the modulation of intestinal macrophages emerged (Table 1): in particular, not only SCFAs and indole derivatives, but also many other diet-derived luminal metabolites, processed by gut microbiota, have been demonstrated to regulate the immune cell functions within the intestine.

#### 3.2.1. Dietary Fiber and Short-Chain Fatty Acids

Dietary fiber is a term used to describe a type of plant-based carbohydrate that, unlike other carbohydrates (such as sugars and starch), is not digested in the human GI tract. Dietary fiber reaches the large intestine relatively intact, serving as an important substrate for the gut microbiota. The process of fermentation employed by the microbes to metabolize the dietary fiber generates the so-called short-chain fatty acids (SCFAs), namely propionate, acetate, and butyrate [47]. These fermentation products promote the growth of specific beneficial microbiota species, such as *Bifidobacteria* and *Lactobacilli*, exert anti-carcinogenic and anti-inflammatory properties (inhibiting NFκB transcription via GPR41) and are able to modulate the immune response in the intestine [48,49]. Recent studies have demonstrated that butyrate is able to modulate gut homeostasis by regulating intestinal macrophage function. Specifically, treatment of macrophages with butyrate leads to the downregulation of LPS-induced pro-inflammatory mediators, including NO, IL-6, and IL-12 [45].

#### 3.2.2. Functional Amino Acids

Dietary amino acid deficiency can cause malnutrition, impairing intestinal immune system and rendering the host more vulnerable to infectious disease. Moreover, these amino acids have been reported to regulate intestinal macrophage functions [50,51]. Even though further studies are needed to mechanistically explain how dietary amino acids can control macrophage activity, it has been demonstrated that (1) they stimulate the replenishment of intestinal macrophages and their IL-10 secretion [51]; (2) arginine, glutamine and tryptophan are able to promote macrophages phagocytic activity [52]; and (3) in *IL-10*^−/−^ mice, histidine inhibits the production of pro-inflammatory cytokines (TNF-α and IL-6), through the suppression of NF-κB activation, thus counteracting colitis development [50,53,54].

#### 3.2.3. Vitamins

Vitamins A and D are both involved in the control of macrophage functions. For instance, retinoic acid (RA), the active metabolite of vitamin A, is produced by intestinal macrophages, and it is crucial to modulate antigen-presenting cell functions within the intestine. It has been reported that a vitamin A-deficient diet can lead to a systemic pro-inflammatory state [55], decrease phagocytic and bactericidal activity of macrophages [56] and favor a non-symptomatic reservoir of *Escherichia coli*-like enteric infections [57]. In agreement with that, supplementation of vitamin A or RA seems to attenuate intestinal inflammation in experimental models [58]. Moreover, RA reduces the synthesis of IL-12 and TNF-α from LPS-stimulated macrophages, while enhancing IL-10 production [59]. Oral administration of RA in vivo inhibits the growth of *Mycobacterium tuberculosis*, downregulating gene transcription of tryptophan aspartate-containing coat protein (TACO) [60], whose transcriptional repression can restrict bacterial entry and survival in human macrophages [61]. On the other hand, vitamin D suppresses the production of pro-inflammatory cytokines in macrophages, via targeting MAPK phosphatase-1 [62], and directly stimulates the expression of the host defense peptide (HDP) cathelicidin, which is required, in macrophages, for the anti-microbial activity against *M. tuberculosis* [63]. Macrophages and neutrophils are major sources of most HDPs: therefore, HDP induction, through vitamin D activity, represents an important mechanism in enhancing macrophage functions [53,54].

## 4. Macrophages in Intestinal Inflammation

### 4.1. Macrophages in Inflammatory Bowel Diseases (IBDs)

**Figure 3:**
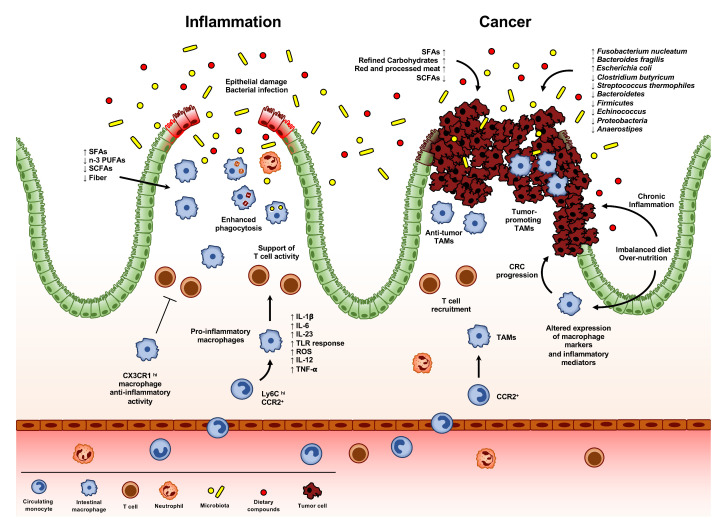
**Intestinal macrophages in inflammation and cancer.** Alteration of intestinal immune homeostasis may result in acute or chronic inflammation. This typically occurs during inflammatory bowel disease (IBDs), damage of the epithelial layer, or infection. Dietary components can exert either beneficial or detrimental effects in these processes, activating a pro-inflammatory response in macrophages, like saturated fatty acids (SFAs), or preventing an inflammatory response, like short-chain fatty acids (SCFAs), fibers, or n-3 polyunsaturated fatty acids (n-3 PUFAs). This state of inflammation is characterized by the recruitment and accumulation of Ly6C^hi^ CCR2^+^ monocytes, which differentiate in pro-inflammatory macrophages, able to secret inflammatory molecules, such as IL-1β, IL-6, IL-23, ROS, IL-12 and TNF-α, to promptly respond to TLR engagement and to support T cell activity. On the other hand, CX3CR1^hi^ macrophages maintain their pro-resolving function. During the resolution of inflammation, intestinal macrophages increase their phagocytic activity, engulfing epithelial apoptotic cells, neutrophils and invading bacteria. The composition of the gut microbiota exerts a crucial role in CRC progression, also impacting on the efficacy or toxicity of many therapeutic agents, including immunotherapies. Differences in the composition of the gut microbiota have been observed between healthy individuals and CRC patients: the presence of bacteria species, such as *Fusobacterium nucleatum, Bacteroides fragilis* and *Escherichia Coli*, has been associated with CRC progression. Conversely, other bacteria, including *Clostridium butyricum*, *Streptococcus thermophiles*, and the species *Bacteroidetes*, *Firmicutes*, *Echinococcus*, *Proteobacteria* and *Anaerostipes* have been negatively associated with the network of butyrate-producing CRC-depleted microbes. In addition, some nutritional constituents, including saturated fatty acids (SFAs), refine carbohydrates and red and processed meat, have pro-inflammatory properties, while short-chain fatty acid (SCFAs) production, as a consequence of microbiota metabolism of dietary fiber, reduces the risk of CRC occurrence, by regulating the intestinal inflammatory response. In addition, overnutrition and imbalanced diet (such as the Western diet), can favor obesity, increasing chronic inflammation and altering the expression of macrophage markers and inflammatory mediators, finally resulting in CRC progression. Tumor-associated macrophages (TAMs) represent the most abundant leukocyte population infiltrating neoplastic tissues. Differently from other tumor types, the role of TAMs in CRC is still controversial. Several studies reported a correlation between higher TAM infiltration and CRC poor prognosis, while other claimed their antitumor activity. The general idea is that the role of TAMs in CRC progression can be influenced by their localization within the tumor tissue: intratumoral macrophages are likely to be more influenced by the tumor cell-generated milieu, thus displaying a protumoral activity; while TAMs localized at the tumor invasive front are less subjected to the conditioning of the tumor-microenvironment, thus exerting anti-tumoral functions. Furthermore, infiltration of T cells at the tumor invasive front has been demonstrated to correlate with better patients’ prognosis.

The alteration of intestinal immune homeostasis may lead to a state of acute or chronic inflammation in the gut. This typically occurs in inflammatory bowel diseases (IBDs), such as Crohn’s disease (CD) and ulcerative colitis (UC), where impaired immune response, in genetically predisposed patients, results in a condition of chronic inflammation of the GI tract, requiring lifelong therapeutic interventions [64]. During IBDs, a clear accumulation of CD14^hi^ CD11c^hi^ macrophages comes to outnumber CD46^+^ HLA-DR^hi^ CD14^lo^ resident macrophages, leading to the formation of a gut microenvironment rich in pro-inflammatory cytokines, such as IL-1β, IL-6, IL-23, IL-12, CCL11, and TNF-α (Figure 3) [65,66]. Accordingly, anti-TNF treatment in IBD patients results in mucosal healing, associated to the reduction of CD14^hi^ macrophages and to an accumulation of their CD206^+^ counterpart, showing pro-resolving activity [67]. Moreover, colonic macrophages in patients with CD show an abnormal morphological maturation, with an altered expression of surface markers and prolonged intracellular bacterial survival [68]. Several studies demonstrated that, in CD patients, also circulating monocytes are characterized by an increased production of pro-inflammatory cytokines, including IL-23 and TNF [69].

Several mouse models of intestinal inflammation have been set up in order to investigate the molecular aspects involved in these processes. As observed in humans, all these models are characterized by a clear accumulation of Ly6C^hi^ monocytes and consequent expression of intermediate levels of CX3CR1. Mirroring what is observed in human IBDs, these cells produce pro-inflammatory mediators, express TREM-1 and high level of ROS, and display a high responsiveness to TLR stimulation (Figure 3) [10]. Despite the presence of a pro-inflammatory milieu, CX3CR1^+^ resident macrophages still retain their anti-inflammatory activity: accordingly, genetic depletion of *Cx3cr1* results in an increased inflammatory condition and tissue damage in a mouse model of DSS-induced colitis [27,28]. Infiltration of Ly6C^hi^ monocytes seems to be crucial for the development of the intestinal pathology: defects in the recruitment of monocytes into the inflamed mucosa, due to the deletion or neutralization of CCL2, CCR2, or β7 integrin pathways, result in a less severe DSS-induced colitis [70,71,72]. Of note, the importance of CCL2-CCR2 axis has been demonstrated also in humans, with a higher infiltration of CCR2^+^ monocytes and increased expression of CCL2 and CCL4 in the mucosa of IBD patients (Figure 3) [73,74].

As in homeostatic conditions, resident macrophages can be involved in the recruitment of monocytes into inflamed mucosa via the secretion of CCR2 ligands: a specific subset of CX3CR1^hi^ CD169^+^ macrophages expand during experimental colitis and influences disease, recruiting monocytes through the production of CCL8 [75,76]. Macrophages also have an effect on other innate and adaptive immune cells: CD14^hi^ macrophages support pathogenic T lymphocyte activity through the secretion of IL-23 and the expression of CD40 and CD80 [69,77,78]. Similarly, macrophages may also induce the recruitment of eosinophils, even though the precise role of this immune cells within the inflamed mucosa is still debated [79,80].

### 4.2. Macrophages in Intestinal Infection

The localization of mucosal macrophages, in closed proximity to the intestinal epithelium, is important for their early recognition of luminal pathogens entering the mucosa and for the initiation of an immediate antibacterial or parasitic response. As previously discussed, CX3CR1^+^ macrophages extend TEDs through the epithelial layer to sample potential pathogens into the intestinal lumen [33,34]. In addition to TED formation, CX3CR1^+^ macrophages are also able to extravasate from the lamina propria directly into the lumen during *Salmonella* infection and phagocyte bacteria to prevent their entry into the tissue [81]. In accordance to the importance of macrophages in the response of intestinal infections, *Ccr2*^−/−^ mice are more susceptible to *Citrobacter rodentium* and *Toxoplasma gondii* infection [42,82]. Interestingly, as *Citrobacter rodentium* infection occurs, macrophages express a series of pro-inflammatory mediators, including IL-1β, IL-23, and TNF-like ligand 1A, stimulating ILC3 to produce IL-22 and leading to the secretion of RegIIIβ and RegIIIγ and bacterial clearance [30,83,84]. Moreover, in a mouse model of intestinal infection by *Helicobacter pylori* and *Citrobacter rodentium*, engagement of macrophage epidermal growth factor receptor results in macrophage activation and cytokine production, thus contributing to tissue inflammation [85]. Besides their pro-inflammatory role during infection, Ly6C^hi^ monocytes can also have regulatory activities: during acute toxoplasmosis, Ly6C^hi^ monocytes secrete PGE2 and IL-10 in response to microbiota, inhibiting neutrophil activation. Accordingly, *Ccr2*^−/−^ mice are more susceptible to this infection, suggesting that monocytes exert multiple functions during mucosal infection [82,86].

In addition to commensal bacteria, the gut hosts also soil-transmitted helminths, especially intestinal nematodes, able to induce tissue damage and requiring the activation of a type 2 cytokine-mediated response [87]. Together with the promotion of Goblet cell activity and the restoration of the mucus layer, type 2 immune response promotes the activation of M2 macrophages, supporting the resolution of inflammation and tissue repair [88,89]. The role of M2 macrophages in helminth infection is still under investigation: while they are necessary for the removal of *Heligomosoides polygyrus*, they seem to be more involved in tissue repair, rather than parasite expulsion, during *Nippostrongylus brasiliensis* infection [90,91,92]. A role of macrophages in tissue repair has been observed also after the elimination of *Trichuris muris*: of note, while the accumulation of macrophages in the type 2 immune response of other tissues is conventionally associated to a local proliferation of resident cells, M2 macrophages increase during *Trichuris muris* intestinal inflammation depends on monocyte infiltration [93,94,95].

### 4.3. Macrophages in Resolution of Intestinal Inflammation

During the resolution of intestinal inflammation, macrophages play a central role in the clearance of bacterial components and apoptotic cells (Figure 3). Upon tissue damage, the innate immune system induces the recruitment of circulating neutrophils that, in turn, attracts monocytes. The first step of resolution process is the apoptosis of neutrophils, whose clearance by macrophages is induced by the expression of a sort of “find me” and “eat me” signals on neutrophils themselves, like CX3CL1, sphingosine 1-phosphate, and lysoposphatidylcoline [96,97,98]. Upon efferocytosis of apoptotic cells, macrophages switch to an anti-inflammatory profile: it has been observed that interruption of DSS administration results in a massive reduction of CX3CR1^int^ macrophages and a concomitant restoration of the CX3CR1^hi^ subset [99]. A number of macrophage-derived pro-resolving mediators have also been identified, including eicosanoids (prostaglandins and leukotrienes), small molecules, proteins, and peptides. For instance, prostaglandin D_2_ (PGD_2_) and its receptor DPI have been detected in long-term remission patients from UC [100]. Accordingly, DPI ablation in myeloid cells results in decrease proportion of pro-resolving macrophages and worsening of the disease in both DSS- and 2,4,6-trinitrobenzenesulfonic acid (TNBS)-induced colitis, indicating a role of PGD_2_ in the resolution of mucosal inflammation [101]. In addition, PGE_2_ has been associated with intestinal resolution of inflammation: it has been demonstrated that the axis PGE_2_-EP4-cAMP enhances the anti-inflammatory activity of macrophages [102]. Accordingly, genome-wide association studies identified a single-nucleotide polymorphism in the gene encoding PGE_2_ receptor in patients with Crohn’s disease [103].

### 4.4. Diet, Microbiota, and Macrophages during Intestinal Inflammation

Mounting evidence indicates that diet exerts a crucial effect on health, both beneficially and detrimentally impacting on microbiota composition and regulating host physiological responses. Both CDs and UCs are multifactorial pathologies also influenced by environmental factors, including dietary habits and microbiota. Hereof, it has been demonstrated that the so-called “Western diet” (WD) is associated with increased risk of developing IBDs [104]. A WD pattern is characterized by a high intake of proteins (mainly from animal-derived sources), saturated fatty acids, refined grains, sugar, alcohol, processed food, and salt, together with a reduced consumption of vegetables, whole grains, vegetable-derived protein sources, fruits, vitamins, minerals, and omega-3 (n-3) polyunsaturated fatty acids (PUFAs) [105]. Together with IBDs, WD pattern has also been significantly associated with the development of non-communicable diseases (NCDs), such as cancer, type 2 diabetes, obesity, metabolic syndrome, and cardiovascular diseases [106,107]. Indeed, mice fed with a WD show a dramatic dysbiosis, with outgrowth of pathobionts and reduction or complete loss of commensal bacteria. This dysbiotic microbiota influences the host’s immune system through several mechanisms, including modification of the signaling via the NLRP6 inflammasome and TLRs, the reduction of AMP and mucus release into the lumen, the degradation of secretory IgAs and the selective loss of IL-10-producing T_reg_ lymphocytes. These changes result in the perturbation of the barrier integrity and in the alteration of intestinal immune cell homeostasis, thus favoring the onset of immune-mediated inflammatory diseases [108,109].

Recently, a critical role for commensal bacteria in the early phase of IBD occurrence has been identified. Moreover, the enteric microbiota of patients with IBDs presents alterations in the microbe balance, with a decrease in *Firmicutes*, *Bacteroidetes* and anti-inflammatory commensals, such as *Faecalibacterium prausnitzii*, an increase in *Enterobacteriaceae*, such as *Escherichia coli* and a frequent identification of adherent and invasive *E. coli* strains [110,111,112].

Notably, specific dietary components are able to impact either positively or negatively on IBD development and/or resolution (Table 1 and Figure 3).

#### 4.4.1. Saturated Fatty Acids (SFAs)

SFAs have been shown to activate a pro-inflammatory response in macrophages through the stimulation of a TLR4-induced inflammatory pathway, which in turn triggers NF-κB and induces the expression of many pro-inflammatory mediators [113]. Moreover, a high intake of SFAs modifies the gut microbiota composition, increasing the proportion of Gram-negative bacteria and altering the intestinal permeability, finally leading to a state of metabolic endotoxemia [114].

#### 4.4.2. N-3 Polyunsaturated Fatty Acids (n-3 PUFAs)

N-3 PUFAs are able to prevent and/or treat different inflammatory diseases, including IBDs. Of note, n-3 PUFAs inhibit TLR4 signaling and the subsequent transcription of pro-inflammatory mediators, as well as activate the anti-inflammatory transcription factor PPAR-γ, thus inhibiting NF-κB-dependent cytokines, including TNF-α [115,116]. Metabolism of the dietary-derived n-3 PUFAs in non-immune and immune cells, including macrophages, results in the formation of a large and growing class of signaling molecules, called specialized pro-resolving mediators (SPMs). Pre-clinical studies, carried out in animal models and human tissues, indicate a role for SPMs in orchestrating the resolution of inflammation. Prominent members include resolvins and protectins: in particular, protectins are able to promote resolution of inflammation, increasing macrophage phagocytosis and reducing pro-inflammatory cytokine release in various diseases, including IBDs [117,118,119]. On the other hand, resolvin D1 ameliorates colitis, suppressing inflammatory macrophages via LXA4 receptor activation. Due to their wide range of positive effects in the resolution of intestinal inflammation, SPMs are being tested in several clinical trials by oral supplementation of n-3 PUFAs, showing beneficial effects in IBD patients [16,120,121,122,123].

#### 4.4.3. Fiber and Short-Chain Fatty Acids (SCFAs)

SCFAs promote the growth of beneficial *Bifidobacteria* and *Lactobacilli* and exert an anti-inflammatory activity on the gut microbiota. Accordingly, reduced levels of SCFAs have been linked to UC conditions [124]. A significant decrease in butyrate-producing bacteria of the Firmicutes phylum was found in UC and CD patients [125,126]. Indeed, some studies have demonstrated that SCFAs administration can mitigate intestinal inflammation and lesions in patients with colitis and in murine models [53,54,127]. Interestingly, some fiber containing food (mainly fruits and vegetables) are rich in bioactive plant-derived phytochemicals, such as quercetin, whose administration, in vitro, was able to suppress LPS-induced and spontaneous inflammation in organoids from, respectively, WT and ulcerative colitis mouse model [128].

#### 4.4.4. Micronutrients

More than half of patients suffering from IBDs (more CDs than UCs) show deficiencies in micronutrients (vitamins and minerals), most commonly vitamins A, B1, B6, B12, D, and K, as well as iron, folic acid, selenium, and zinc. This deficit is thought to be caused by dysbiosis, damaged host mucosal system and malabsorption [129]. Gut commensal microbiota is crucial to guarantee proper production and bioavailability of some vitamins [130]. Vitamin D deficiency has been linked to both extended illness duration and higher disease activity in IBD patients. Moreover, some human polymorphisms in the vitamin D receptor (VDR) are associated with IBD susceptibility [131]. VDR-targeted pathways include TLR, NF-κB signaling, and Th17/T_reg_ cell response: as NFκB-induced pathways are enhanced in VDR-deficient mice exposed to bacterial and chemically-induced colitis, it has been proposed that the anti-inflammatory function of vitamin D depends on the suppression of NFκB activity [132]. Vitamin D effect has also been linked to the modification and control of the gut microbial composition: *VDR*^−/−^ mice show an altered microbiota, more abundant in Bacteroidetes and Proteobacteria then in Firmicutes phyla. Interestingly, vitamin D administration to mice infected with *Citrobacter rodentium* results in an increased pathogens burden: specifically, vitamin D treatment leads to a spoiled Th17/T_reg_ cell response, which is essential for *C. rodentium* clearance, thus impairing mucosal host defense against an enteric bacterial pathogen [53,133,134]. Moreover, vitamin K has been shown to exert a protective effect against DSS-induced colitis in mice, through the inhibition of inflammation via IL-6 suppression in B cells [135]. Moreover, zinc and selenium deficiencies, typically occurring in IBD patients, may contribute to the persistent inflammatory process caused by the disease. Preliminary data reported that zinc supplementation can ameliorate mucosal barrier dysfunction, by modulating tight-junction proteins in both the small and large intestine [136].

#### 4.4.5. Impact of Overweight on IBDs

The WD pattern results in high-calorie uptake, a subsequent increase of adipose tissue and a rapid bodyweight increase. This diet is associated with elevated serum markers of inflammation, suggesting either direct or indirect stimulation of the immune system. In general, the adipose tissue is now considered as an active endocrine organ, containing both adipocytes and immune cells (T cells and macrophages), able to produce hormones, such as adipokines (leptin and resistin, with pro-inflammatory activities) adiponectin (with anti-inflammatory activities), cytokines, and chemokines. Interestingly, the adipose tissue in lean mice is characterized by an anti-inflammatory profile, with M2-polarized macrophages and T_reg_ lymphocytes secreting IL-4, IL-10, IL-33, and adiponectin. On the contrary, obese mice show a pro-inflammatory profile, with M1 macrophages secreting TNF-α, IL-1β, and IL-6 cytokines [137]. Even though a correlation between overweight and/or obesity and IBDs is not yet confirmed, the adipose tissue in CD patients shows a strongly pro-inflammatory cytokine profile [138]. Moreover, several features of the CD adipocytes, such as TLRs expression, higher presence of commensal bacteria, augmented translocation of intestinal bacteria, and increased C-reactive protein production, suggest that they are involved in the antimicrobial response, working as a barrier to maintain gut homeostasis and linking the adipose tissue with the innate immune responses [139]. Finally, the enlarged mesenteric tissue wrapped around the intestine, a typical feature of patients with CD, is usually found adjacent to inflammatory lesions: this so called “creeping fat” correlates with disease activity and is characterized by high infiltration of lymphocytes and macrophages. Altogether, these findings illustrate that mesenteric obesity may be involved in CD pathogenesis [53].

In contrast to WDs, traditional dietary patterns, such as the Mediterranean, the Japanese, and the vegetarian diet, not only reduce NCDs burden, but also negatively correlate with serum markers of inflammation and exert beneficial effects on the gut microbiota. Interestingly, all those diets share several common healthy features, such as a high intake of vegetables, whole grains, fruits, vegetable-derived protein sources, fish, unsaturated fats (with a high mono- and polyunsaturated-to-saturated fat ratio and n-3 PUFAs rich), together with a low or no consumption of red and processed meat, simple sugars, saturated fats, processed, and packaged foods [140,141,142,143].

Different kind of diets, dietetic components and body composition are able to influence the health status, either beneficially or detrimentally, mounting an anti- or a pro-inflammatory response in the gut, thus impacting on intestinal macrophages homeostasis and microbiota composition.

## 5. Macrophages in Colorectal Cancer (CRC)

### 5.1. Tumor-Associated Macrophages (TAMs)

Inflammatory cells are key components of the tumor microenvironment and macrophages represent the most abundant leukocyte population infiltrating neoplastic tissues [144,145,146]. Tumor-associated macrophages (TAMs) mainly originate from BM precursors, recruited into cancer tissue by chemokines (e.g., CCL2, CCL5, and CXCL12), growth factors (e.g., CSF1) and products of the complement cascade, although local proliferation has been demonstrated in some tumors [147]. Although highly schematic, macrophages can be conventionally classified into two functionally different subsets, defined as “classically activated”, or M1, and “alternatively activated”, or M2, macrophages [148,149,150]: indeed, M1 and M2 phenotypes represent the extremes of a continuum spectrum of different phenotypic features, which are determined by the stimuli coming from the surrounding environment. M1 polarization is driven by Th1 type cytokines (e.g., IFN-γ) or bacterial product, and M1-polarized macrophages classically produce pro-inflammatory mediators, have bactericidal activity and support Th1-type immune response. Conversely, M2 polarization is induced by Th2 cytokines (e.g., IL-4 or IL-13) and immunosuppressive stimuli (e.g., IL-10 and TGF-β), and M2-polarized macrophages have immune-regulatory functions, being source of IL-10, arginase, and TGF-β, maintain tissue homeostasis and are involved in tissue remodeling and wound healing processes [151,152,153]. In nascent neoplasia, M1-macrophages can kill tumor cells, contributing to the “elimination” phase of immune-editing process. Along with cancer progression, following the “equilibrium” and “escape” phases of cancer immune-editing, tumor microenvironment elicits the alternative, M2, activation of TAMs, induced by the secretion of cytokines, such as IL-4 and IL-13, by Th2 cells, basophils, and eosinophils [144]. Furthermore, tumor cells themselves, B cells, and stromal cells can promote phenotypic shifts in macrophages, not fitting to classic M1-M2 dichotomy previously described, resulting in a variety of tumor-dependent heterogeneous TAM phenotypes and functions [146]. Although phenotypic plasticity and diversity of TAMs have been extensively described, they have been generally associated with poor prognosis in different tumor types [154,155,156]. In growing cancer, TAMs express peculiar surface molecules, including CD163 and CD206, and display features related to angiogenesis, lympho-angiogenesis and tissue remodeling, thus favoring tumor progression and metastasis formation [146,157]. Overall, TAMs contribute to the formation of an immunosuppressive tumor microenvironment, by secreting immune-regulatory cytokines, including IL-10 and TGF-β, inducing T lymphocyte starvation and triggering the inhibitory PD-1-mediated immune checkpoint in T cells [144,158,159].

### 5.2. TAMs and CRC

Colorectal cancer (CRC) represents the third cause of cancer-related death in Western countries. More than 70% of CRC are sporadic tumors, occurring as the results of genetic alterations arising to colonic epithelial cells and driving the transformation of normal epithelium to adenomatous polyps and invasive cancer [160]. Several risk factors, including obesity, alcohol assumption, processed meat intake, smoke, lack of physical activity, hypertension, and abnormal blood lipids, have been associated with CRC onset [161,162]. On the other hand, a proportion of CRC can have a hereditary origin, such as the familial adenomatous polyposis (FAP) and the hereditary non-polyposis colorectal cancer (HNPCC) [163,164]. CRC represents a paradigm of the cancer-related inflammation concepts: it has been extensively reported that IBD patients, both UCs and CDs, show an increased risk to develop colitis-associated cancer (CAC) [160,165].

Independently from its origin, the inflammatory microenvironment plays a crucial role in the onset and progression of CRC: tumor cells secrete a variety of inflammatory mediators acting on immune infiltrating cells, endothelial cells and fibroblasts, leading to the formation of a tumor-promoting microenvironment rich in cytokines, chemokines, innate immunity receptors, and signaling molecules [166,167]. A leukocyte infiltration, mainly composed by T-lymphocytes and macrophages, has been already observed in the early phases of CRC, strongly increasing with tumor growth. As for other tumors, T cell recruitment, especially along the tumor invasive front, has been associated with a good prognosis in CRC patients [168,169,170]. In the same direction, neutrophil infiltration has been correlated with a good prognosis and better response to 5-FU-based chemotherapy [171].

Differently from other tumor types, the role of TAMs in CRCs is still controversial (Figure 3) [167,172]. Genetic inactivation of STAT3 in macrophages has been correlated with chronic inflammation and intestinal tumor development [173]. In line with this, macrophage infiltration and tumor load in a *Ccr2*^−/−^ mouse model of CAC was significantly reduced [174]. In the same mouse model, a proteomic analysis revealed a different ECM signature in TAMs, with up to 46 genes differently expressed in WT and *Ccr2*^−/−^ mice, showing a higher tumorigenic potential of TAM-positive ECM [175]. Additionally, ECM from human colorectal cancer has been shown to educate macrophages towards an M2 phenotype in the tumor microenvironment [176]. Differently, genetic deletion of *Cx3cr1* results in an increased occurrence of CAC, due to lower level of HMOX-1, a crucial anti-inflammatory and antioxidant enzyme produced by different cells, including macrophages. Of note, in *Cx3cr1*^−/−^ mice, AOM-DSS treatment results in a different microbiota composition compared to WT counterpart, together with a reduced β-diversity and an altered expression of *Akkermansia muciniphila* [177]. Evidence of tumor cell killing activity of TAMs has been demonstrated in a rat model of colon carcinoma: although peritoneal macrophage depletion results in better differentiated tumors with decreased vascularization, macrophage-depleted rats have a worse prognosis, with higher tumor load and reduced survival [178]. Differently, targeting CCR2 in a mouse model of colorectal cancer liver metastasis reduces TAM accumulation at the metastatic site and restores antitumor immunity [179,180].

The same controversial role of TAMs in CRC progression has also been found in humans. Some clinical and epidemiological studies indicate that TAM infiltration is associated with advanced tumor stage and worse prognosis, while others correlate with improved survival and reduced liver metastasis [181,182,183,184]. In particular, macrophage infiltration at the tumor front has been positively associated with a better prognosis, as macrophage-to-tumor cell contact is necessary for their anti-tumorigenic activity [181]. Accordingly, high TAM density at the tumor invasive front correlates with lower incidence of hepatic metastasis and improved patient prognosis [182]. In contrast, counting macrophages in the entire tumor area was not considered a good prognostic indicator [185]. Recently, in stage III CRC, higher CD68^+^ macrophage infiltration has been correlated with decreased overall survival; on the contrary, another study showed that CD68^+^ macrophage infiltration at the tumor invasive front associates to increased overall survival in patients treated with 5-FU [186,187]. Interestingly, it has been demonstrated that macrophage subpopulations differently distribute between the invasive front, internal tumor area or adjacent normal mucosa: CD68^+^ cells are located more in the tumor area, while CD80^+^ macrophages are highly expressed within the adjacent normal mucosa. Moreover, an association between higher CD80^+^/CD163^+^ cell ratio at the tumor invasive front and improved survival was found, as well as a protective role of CD80^+^ macrophages in preventing tumor relapse [187].

Overall, these data suggest that the role of TAMs in CRC progression may be strongly influenced by their localization within the tumor tissues: macrophages at the tumor invasive front are less subjected to the conditioning of the tumor microenvironment and of hypoxic area, thus exerting an antitumoral, rather than pro-tumoral, activity. Similarly, at the early tumor stage, macrophages could be more effective in killing neoplastic cells, while, in the advanced tumor stage, they are likely shifted to a M2 pro-carcinogenic phenotype. The identification of the different macrophage subsets, having different functions, is crucial to evaluate their role in CRC progression and, consequently, to consider their use as possible predictive or prognostic biomarkers.

### 5.3. Diet, Microbiota and Macrophages in CRC

The third expert report by the World Cancer Research Fund (WCRF) and the American Institute of Cancer Research (AICR) indicates that some nutritional constituents, such as saturated fats, refined carbohydrates, and red and processed meat, own pro-inflammatory properties and, together with obesity and low physical activity, can be considered some of the most significant exogenous factors in CRC etiology. Accordingly, approximately 50% of CRC cases in the USA and UK were estimated to be attributable to the above-mentioned modifiable risk factors [161]. Besides that, dietary constituents exert a huge impact on the host gut microbiota homeostasis, acting on tissue inflammation, cancer initiation and/or progression, as well as altering immunological and inflammatory parameters of gut microbiota composition [188]. Several studies have identified differences in the compositions of intestinal microbiota between patients with CRC and healthy individuals. Moreover, mounting evidence demonstrates that changes in the gut microbiota occur during the early stages of colorectal carcinogenesis and can be used as biomarkers for the early detection of CRC [189]. Moreover, it can also impact on the efficacy or toxicity of many therapeutic agents, including immunotherapies [190].

Numerous meta-analyses have found consistent associations of CRC with several bacteria, across different human populations [191]. Interestingly, *Fusobacterium nucleatum* has been linked to advanced stage of the disease, higher risk of recurrence and shorter patient survival [192]. In addition, *Bacteroides fragilis* consistently increased in the microbiota of patients with CRC, also in the advanced state, while *Escherichia coli* has been found to be enriched both in IBD and CRC patients, compared with healthy individuals [193,194]. On the contrary, the presence of other bacteria, such as *Clostridium butyricum*, the lactic acid bacterium *Streptococcus thermophiles* and the species *Bacteroidetes*, *Firmicutes*, *Echinococcus*, *Proteobacteria* and Anaerostipes negatively correlate with the network of butyrate-producing CRC-depleted microbes (Figure 3) [195,196,197,198].

Preliminary data indicate that some dietary components and interventions can exert an anti-tumorigenic effect, modulating macrophage activities. Interestingly, in Apc^Min+^ mice, quercetin, which exerts a well-documented anti-carcinogenic effect in vitro, has been shown to decrease polyp number and size, via the reduction of macrophage infiltration in the intestinal villi [199]. Moreover, alternate day fasting, in mice, inhibits colon carcinoma cell growth, without causing a reduction of body weight, suppressing M2 polarization and TAM proliferation through the inactivation of JAK1/STAT3 signaling pathway [112].

Mounting evidence has shown that specific nutrients can exert a relevant impact on CRC development or prevention (Table 1 and Figure 3).

#### 5.3.1. Red and Processed Meat

The WCRF report demonstrated that each 100 g/day increase in intake of red and processed meat was associated with a 12% higher risk of CRC [161]: this effect might be mediated by the intake of preservatives in red and processed meats (such as nitrates and nitrites), carcinogenic chemicals produced during meat processing and cooking (namely, heterocyclic amines and polycyclic aromatic hydrocarbons), and nutrients enriched in meats (like heme iron, choline and carnitine) [161,200]. Some of these components can be metabolized by the gut microbiota and, in turn, produce metabolites that have been associated to CRC development. Of note, red meat has a high content of choline and carnitine, which are precursors to the gut microbiota-mediated formation of trimethylamine (TMA) and trimethylamine N-Oxide (TMAO). Chronic ingestion of red meat causes an increase in urine and plasma levels of TMAO, whose pathway has been implicated in the development of CRC [201]. Despite data supporting this hypothesis in humans are weak, it seems that a higher saturated fat content, characteristic of processed meat, may stimulate tumorigenesis through the synthesis of peculiar secondary bile acids [161]. Bile acids secreted by liver, in fact, can be deconjugated by bacterial microbiota to produce two secondary bile acids (lithocholic and deoxycholic acid), which, at elevated concentrations, may contribute to carcinogenesis, via a pro-inflammatory activity [202].

#### 5.3.2. Fiber and Short-Chain Fatty Acids (SCFAs)

Several publications have demonstrated an association between fiber intake and reduced risk of CRC [161,203,204]. The WCRF reports that an increase in whole grains consumption of 90 g/day caused a 17% reduction in CRC risk [161]: this effect could be related to the influence of fiber on the gut microbiota, with a consequent SCFA production that, in turn, reduces the risk of CRC by regulating metabolism and inflammatory response, through the modulation of different immune cells, including macrophages. Fiber-containing foods are also rich in bioactive plant-derived phytochemicals that, in preclinical studies, exert chemopreventive effects on CRC. However, their impact on CRC development, in humans, is still under investigation [205].

#### 5.3.3. Impact of Overweight on CRC

The WCRF reported that each 5 kg/m^2^ increase in body mass index (BMI) is associated with 5% rise in CRC risk [161]. Overnutrition and imbalanced diets, such as WD, can favor overweight and obesity, thereby increasing chronic inflammation, which is notably a significant cancer risk factor. Adipose tissue is highly infiltrated by immune cells, including NK cells, mast cells, neutrophils, DCs, and classically activated macrophages, able to mount a local inflammatory response, which can finally promote tumor progression [206]. Macrophages, which in lean people show an M2 anti-inflammatory phenotype, during obesity are switched to an M1 pro-inflammatory profile, expressing tumor-promoting cytokines, such as TNF, IL-6, IL-1β and chemokines, such as CCL2 and MIF [207,208]. In particular, in a mouse model of intestinal tumorigenesis, the administration of a high fat diet (HFD), which mimics the macronutrient content of a standard American WD, altered the expression of macrophage markers and inflammatory mediators within adipose tissue and tumor microenvironment, therefore increasing adiposity and enhancing CRC progression [209]. Another study showed that chemically-induced CAC is exacerbated by diet-induced obesity: in this context, IL-6 production stimulates macrophage polarization to a tumor-promoting phenotype, which prompts CAC development [210].

## 6. Targeting Intestinal Macrophages in Inflammation and Cancer

Accumulating evidence suggests the idea that the stimulation of the pro-resolving phenotype in macrophages could be considered as a novel approach for the treatment of intestinal inflammation. Indeed, most of the classic IBD therapies, including mesalazine and infliximab, have shown to affect macrophage activities, inhibiting inflammatory pathways or inducing an alternative polarization [67,211]. Corticosteroid administration, for example, represents the main treatment for patients with moderate or severe relapse of UCs and CDs: they exert pleiotropic effects on macrophages, promoting monocyte differentiation into M2-macrophages and stimulating efferocytosis [212,213,214,215]. In addition, 5-aminosalitylates show a direct inhibition of NF-κB activation, mainly detected in inflammatory macrophages from UC patients [211]. Treatment with anti-TNF agents is extremely effective in IBD patients, stimulating a regulatory phenotype in macrophages: accordingly, infliximab administration induces CD68^+^ CD206^+^ regulatory macrophages [67]. Other approaches are currently under investigation to enhance a regulatory phenotype in intestinal macrophages. For example, inhibition of phosphodiesterase 4 (PDE4), by apremilast and roflumilast administration, results in the induction of anti-inflammatory mediators: of note, patients treated with apremilast showed clinically significant amelioration of UC symptoms and mucosal healing [16]. Other studies are aimed at using phagocytic capacity of macrophages to deliver therapeutic drugs into inflamed tissue, reducing systemic side effects: this approach has been, indeed, considered for TAM targeting, trying to revert their protumoral activity into the antitumoral phenotype [216].

As previously discussed, the prognostic significance of TAMs in CRC progression is still controversial, mainly due to the different localization of macrophages within tumor tissue and the markers used for their identification. In addition, TAM infiltration can influence the efficacy of therapeutic treatments, such as chemotherapy and radiotherapy: in CRC, high TAM density was independently associated with better disease-free survival only in 5-FU treated patients [144,186]. Another study reported that TAMs get activated during 5-FU treatment and are responsible for the development of CRC chemoresistance [217]. Resistance to oxaliplatin represents another limiting factor in the efficacy of CRC therapy: it has been observed that mice treated with oxaliplatin showed a decrease in M1-macrophage tumor infiltration. Interestingly, administration of the Toll-like receptor agonist R848, in combination with oxaliplatin, was able to revert the macrophage orientation toward an M1-phenotype, enhancing oxaliplatin antitumor efficacy [218]. In addition, two IgG FcγR polymorphisms in metastatic CRCs treated with cetuximab may be used as molecular markers to predict patient clinical outcome, indicating a role of cetuximab in the antibody-dependent cell-mediated cytotoxicity [219]. Recently, it has been reported that the pharmaceutical inhibition of CCR5 induces an anti-tumoral repolarization of TAMs in metastatic CRC: of note, CCR5 blockade induces a STAT3/SOC3-mediated phenotype switch in TAMs, together with a reduction of CD163^+^ cells and a reshape of myeloid cell composition in the tumor microenvironment. This anti-tumoral effect has been also confirmed in a phase I trial in advanced refractory CRC with metastatic liver [220].

In general, macrophage therapeutic approaches are under consideration for CRC therapeutic purpose, aimed at controlling macrophage recruitment and phenotype within tumor tissue [162,172]. Of note, TAM-targeting strategies can be per se beneficial, even if they result to be more effective when used as complementary approach to conventional cyto-reductive, antiangiogenic, or immunomodulating therapies [144,221].

## 7. Conclusive Remarks

In the last years, significant advances have been made in understanding intestinal macrophage immunobiology. The development of single-cell sequencing technologies and fate-mapping approaches has led to the identification of new subtypes and different activation profile of intestinal macrophages, both in physiological and pathological conditions. Similarly, deeper analysis of the gut microbiota has provided an additional environmental cue able to influence macrophage phenotype and functions: in this context, the quality, the kind, and the amount of nutrients in the diet are able to affect the immune system and, importantly, to regulate the gut microbiota. Additional studies are needed to fully define the contribution of gut macrophages in the pathogenesis of intestinal diseases. If a defect in the transition of monocytes to pro-resolving mature macrophages seems to be a crucial step in IBD development, the role of macrophages in CRC is still controversial, strongly depending on TAM localization within tumor tissue and on the markers used for the identification of their phenotype. Additionally, a balanced and healthy nutritional regimen could be employed not only as a form of CRC chemoprevention, but also, when properly structured and individualized by a professional nutritional counseling, to ameliorate the diagnosis after the onset, the life quality after treatment and the adverse effects of chemotherapy and radiotherapy. Antitumor treatments strongly influence the profile of TAMs, which, in turn, are able themselves to support or counteract the effects of these therapeutic interventions. Given the growing interest on macrophages as therapeutic targets, it is critical to better understand the mechanisms controlling their switch from pro-resolving to pro-inflammatory phenotype and vice versa, in order to take advantage of their fine-tuned immunological capability and pharmacologically modulate their status to influence patients’ clinical outcome. Moreover, further studies are needed to deeply understand the mechanisms by which specific dietary nutrients can exert their anti- or pro-cancer effects, affecting macrophage activities and modulating the gut microbiota. These progresses are long expected, in order to get a new class of targets to better treat intestinal inflammatory and neoplastic diseases.

## Figures and Tables

**Table 1:** Specific anti- or pro-inflammatory effects of diets, dietary compounds and body composition on intestinal macrophages and microbiota.

Diets, Dietary Compounds and Body Composition	Anti-Inflammatory	Pro-Inflammatory	Microbiota	Mechanisms
Western diet (WD)	-	↑	Microbiota dysbiosis, with perturbation of barrier integrity and alteration of intestinal immune cell homeostasis [110,111].	Signaling modification via the NLRP6 inflammasome and TLRs; degradation of secretory IgAs and selective loss of IL-10-producing T_reg_ lymphocytes [110,111].
Mediterranean, Japanese and Vegetarian diets	↑	-	Beneficial effects on gut microbiota [142,143,144,145].	Decrease serum markers of inflammation [142,143,144,145].
Saturated fatty acids (SFAs)	-	↑	Gram-negative bacteria increase and intestinal permeability alteration [116].	Pro-inflammatory response activation in macrophages [115].
N-3 Polyunsaturated Fatty Acids (n-3 PUFAs)	↑	-	-	Inhibition of pro-inflammatory mediator transcription; anti-inflammatory transcription response activation [117,118]; resolution of inflammation, increase macrophage phagocytosis and reduction of pro-inflammatory cytokine via specialized pro-resolving mediators (SPMs) [119,120,121].
Fiber and Short-Chain Fatty Acids (SCFAs)	↑	-	Promote the growth of *Bifidobacteria* and *Lactobacilli*; exert an anti-inflammatory activity on the gut microbiota [126].	Anti-carcinogenic and anti-inflammatory properties (inhibiting NFκB transcription via GPR41); immune response modulation in the intestine [49,50]. Down-regulation of LPS-induced pro-inflammatory mediators, including NO, IL-6, and IL-12 [46].
Quercetin	↑	-	-	Suppress LPS-induced and spontaneous inflammation in organoids from, respectively, WT and ulcerative colitis mouse model [130].
Vitamin A and Retinoic Acid (RA)	↑	-	Oral administration of RA inhibits the growth of *Mycobacterium tuberculosis* [61]. Vitamin A deficient diets favor a non-symptomatic reservoir of *Escherichia coli*-like enteric infections [58].	Attenuate intestinal inflammation in experimental models [59]; RA reduces the synthesis of IL-12 and TNF-α from LPS-stimulated macrophages, while enhancing IL-10 production [60].
Vitamin D	↑	-	Control of the gut microbial composition [54,135,136].	Suppression of NFκB activity [134].
Vitamin K	↑	-	-	Inhibition of inflammation via IL-6 suppression, in B cells of DSS-induced colitis mice [137].
Adipose tissue in obesity	-	↑	-	Pro-inflammatory cytokine profile [140]; high infiltration of lymphocytes and macrophages [54].

## Abbreviations

CCR	chemokine receptor
CCL	chemokine ligand
Ly6C	Lymphocyte antigen 6 complex
MHC	Major Histocompatibility Complex
LFA-1	Lymphocyte function-associated antigen 1
VLA-1	Very Late Antigen-1
Tim4	T cell immunoglobulin mucin receptor 4
IL-	Interleukin-
iNOS	inducible nitric oxide synthase
CSF1	colony stimulating factor 1
CSFR1	colony stimulating factor receptor 1
Lrg5	Leucine-rich repeat-containing G-protein coupled receptor 5
STAT3	Signal transducer and activator of transcription 3
TREM1	Triggering Receptor Expressed On Myeloid Cells 1
STAT1	Signal transducer and activator of transcription 1
ILC3	Type 3 innate lymphoid cells
TGFβ	Transforming growth factor β
TNΦ	Tumor necrosis factor
LPS	Lipopolysaccharide
RORγt	Retinoic acid receptor-related orphan receptor γt
CSF2	Colony stimulating factor 2
NO	Nitric oxide
NFκB	Nuclear factor kappa-light-chain-enhancer of activated B cells
GPR41	Free fatty acid receptor 3 (FFA3)
MAPK	Mitogen-activated protein kinase
HLA-DR	Human Leukocyte Antigen – DR
RegIII	Regenerating islet-derived protein 3
PGE2	Prostaglandin E2
TNBS	2,4,6-Trinitrobenzenesulfonic acid solution
EP4	Prostaglandin E2 receptor 4
cAMP	Cyclic adenosine monophosphate
NLRP6	NOD-like receptor family pyrin domain containing 6
PPAR-γ	Peroxisome Proliferator Activated Receptor γ
LXA4	Lipoxin A4
IFN-γ	Interferon-γ
PD-1	Programmed cell death protein 1
5-FU	5-Fluorouracil
ECM	Extra-cellular matrix
WT	Wild-type
HMOX-1	Heme Oxygenase 1
AOM	Azoxymethane
NK	Natural killer
DC	Dendritic cell
MIF	Macrophage migration inhibitory factor
IgG	Immunoglobulin G
FcγR	Fcγ receptor
SOC3	Suppressor of cytokine signaling 3
ROS	Reactive Oxygen Species
